# Patient-Reported Treatment Satisfaction with Rivaroxaban for Stroke Prevention in Atrial Fibrillation. A French Observational Study, the SAFARI Study

**DOI:** 10.1371/journal.pone.0166218

**Published:** 2016-12-09

**Authors:** Olivier Hanon, Edouard Chaussade, Pierre Gueranger, Elise Gruson, Sabrina Bonan, Alain Gay

**Affiliations:** 1 APHP Hôpital Broca, Service de Gériatrie, Paris, France; 2 Université Paris Descartes, Sorbonne Paris Cité, Paris, France; 3 CH La Chartreuse, Service de Cardiologie, Villefranche de Rouergue, France; 4 Bayer Healthcare SAS, Loos, France; 5 ICTA Project Management, Fontaine-les-Dijon, France; University of Bologna, ITALY

## Abstract

**Background:**

For antithrombotic treatments, Patient Reported Outcomes (PRO) and patient satisfaction with treatment are essential data for physicians because of the strong relationship between patient satisfaction and adherence to treatment. The impact of rivaroxaban on patient satisfaction and quality of life was not sufficiently documented in phase III studies. There is a need for further data in this field especially in real life conditions.

**Methods:**

The SAFARI study is composed of patients with non-valvular atrial fibrillation (AF), previously treated with vitamin K antagonist (VKA) and switched to rivaroxaban. Patient satisfaction with anticoagulant therapy was measured by the Anti-Clot Treatment Scale (ACTS), a validated 15-item patient-reported scale including a 12-item ACTS Burdens scale and a 3-item ACTS Benefits scale. Satisfaction of medication was compared between baseline and 1, 3 and 6 months.

**Results:**

Study population was composed of 405 patients. Mean age was 74.8 (standard deviation = 9.0) years and 63.0% were male. Mean CHA_2_DS_2_-VASc score was 3.4 (1.5) and mean HAS-BLED score was 2.9 (1.0). After 3 months of treatment with rivaroxaban, patient satisfaction improved compared with VKA: mean ACTS burdens scores significantly increased by 8.3 (8.9) points (p<0.0001) and ACTS benefits scale by 0.4 (2.9) (p<0.001). Compared with baseline, the improvement in ACTS burdens and benefits became apparent at 1 month (46.5 vs. 53.6 p<0.001 and 10.4 vs. 10.7, p<0.05 respectively) and persisted at 6 months (46.5 vs. 54.76 p<0.001 and 10.4 vs. 10.8 p = 0.02 respectively). Rivaroxaban persistence was 88.7% at 6 months.

**Conclusions:**

SAFARI data support a good risk-benefit balance for rivaroxaban, with a good safety profile and encourage PRO design studies. The switch from VKA to rivaroxaban improved patient satisfaction at 1, 3 and 6 months after rivaroxaban initiation among patients with AF, particularly in reducing patient-reported anticoagulation burden.

## Introduction

The prevention of ischemic stroke due to atrial fibrillation (AF) is based on oral anticoagulants (OAC) [1]. Vitamin K antagonists (VKAs) have been used as a long-term anti-coagulant therapy for more than 50 years. Although the efficacy of VKA is well-established, VKA monitoring is difficult because they have a large inter and intra-individual variability of efficacy and their therapeutic range is very narrow. VKA monitoring relies on regular measurement of the International Normalized Ratio (INR). However even with a regular INR monitoring, a lot of patients are outside the therapeutic range a significant amount of the time [2] that can lead to serious outcomes either thrombosis or bleeding. Moreover because of the regular blood draw needed for the INR measurement, an important proportion of patients discontinue VKA therapy [3]. However optimal stroke prevention with VKA depends not only on tight control of INR but on medication adherence and even more on treatment persistence [4]. New OAC, including rivaroxaban have been shown to be at least as effective and safe as VKA in large randomized controlled trials [5–7], with a significant lower incidence of intracranial hemorrhage, and have now been incorporated into guidelines [1,8], with gradual uptake of their prescription in routine practice [4]. Rivaroxaban is a potent selective direct Factor Xa inhibitor prescribed at a fixed dosage that does not require regular monitoring. The expected advantage of rivaroxaban, therefore, is its greater ease of use compared to VKAs, which may be associated with improved patient satisfaction.

The purpose of the present study was therefore to demonstrate in everyday practice whether use of rivaroxaban was associated with better patient satisfaction compared with VKA in patients with AF after a switch from VKA to rivaroxaban.

## Methods

SAFARI study is a French multicenter, prospective, observational study including patients with non-valvular AF who had undergone a switch of anticoagulant treatment from VKA to rivaroxaban.

### Centers

Hospital-based and private cardiologists located in metropolitan France were offered to participate in the study, and 51 cardiologists (74.5% office-based) participated in the study.

### Study population

Only adult patients with AF already treated with VKA and who intended to start treatment with rivaroxaban were eligible for inclusion. In order to avoid selection bias and to achieve a cohort for whom treatment was in accordance with common daily practice, patients were included in a consecutive manner at each site and no exclusion criteria were defined except patient presenting with at least one of the contraindications listed in the summary of product characteristics. Data were collected at 4 visits: at inclusion (baseline), 1 month, 3 months and 6 months after inclusion (follow-up). Patients were enrolled from April 2013 to June 2014.

### Outcomes

#### Primary endpoint

Primary evaluation criterion was changes in patient satisfaction measured by the Anti-Clot Treatment Scale (ACTS) score from baseline and 3 months. Patient satisfaction with treatment was also measured at 1 and 6 months. The ACTS is a validated 15-item scale developed specifically to evaluate patient’s satisfaction with an anti-coagulant treatment and has been previously used [9]. It includes a 12-item burdens scale and a 3-item benefits scale. In addition one global question is collected for both burden and benefit.

The ACTS Burdens is reverse coded on a 5-point Likert scale from ‘Extremely’ (coded 1) to ‘Not at all’ coded (5). The ACTS Benefits is normally coded from 1 to 5 with higher scores indicating greater satisfaction with treatment. ACTS Burdens score is the sum of the 12 items and ranges from 12 to 60 and ACTS Benefits score is the sum of the 3 items and ranges from 3 to 15.

#### Secondary endpoints

Secondary evaluation criteria were (1) change from baseline in quality of life assessed using the validated SF-36 questionnaire at 1, 3, 6 months, (2) treatment continuation rate, (3) drugs adherence according to patients’ interview (do you forget your treatment: never, rarely, sometimes, frequently or all the time) (4) satisfaction of physicians about the treatment (very unsatisfied, unsatisfied, neutral, satisfied, very satisfied).

Safety data were also recorded by the physician during the follow up of the study.

### Data collection

Data were entered by investigators or their staffs on paper case reports forms (CRF). The ACTS and the SF-36 questionnaires were self-administered by the patients. Independent double-entry of data was performed by two data entry operators. At the end of double entry, discrepancies were corrected by rechecking the forms (CRF or questionnaire) to obtain a clean database.

Quality assurance measures included automated plausibility checks upon data entry, queries based on a validation plan, and on-site quality review in sites presenting issues with a check of CRF data against patient files. Study management was performed by the contract research organization ICTA PM, Fontaine-les-Dijon, France, on behalf of the sponsor, Bayer HealthCare SAS.

### Statistical methods

The ACTS was scored in accordance with the developers’ guidelines [10]. The score for each item was obtained by means of a 5-response Likert rating scale (1 –Not at all, 2 –A little, 3 –More or less, 4 –A lot, 5 –Extremely). If more than 50% of the ACTS questions were incomplete, the scale was taken as missing. The statistical analysis was performed using SAS statistical package version 9.2. Only patients fulfilling the inclusion/exclusion criteria were taken into account for analysis. All variables collected in the CRFs and questionnaires and all derived parameters were used in the descriptive statistical analysis. Numerical data were summarized by mean, standard deviation (SD)). The two-sided 95% confidence interval (CI) of the mean was provided for all primary outcome variables. Binary, categorical, and ordinal parameters were summarized by percentage and numbers within the various categories.

The baseline ACTS scores (burdens and benefits) and ACTS scores after 1, 3 and 6 months were compared using a Wilcoxon signed ranks test (because of non-Gaussian distribution). The hypotheses of the normality of the distribution of change in ACTS scores were verified with the Shapiro-Wilk test. Plots of questionnaire subscale score means by visits were drawn. In addition, a mixed model for repeated measure (MMRM) was used to assess whether ACTS scores were different between age groups across the visits (baseline, month 1, month 3 and month 6) throughout the whole study period.

The clinical significance of observed mean difference over time was interpreted in terms of equivalents effect size, calculated as the mean difference in scores at time point 1 to time point 2 divided by the standard deviation of the time 1 score [11]. Effect size was interpreted as the following: 0.2 (small change), 0.5 (moderate change), and 0.8 (large change) [11,12].

The persistence to rivaroxaban (cumulative treatment continuation rate) was assessed using a Kaplan-Meier analysis during a 180-day follow up period. Patients who withdraw from the study while they were still treated with rivaroxaban were censored at their last contact.

The patient adherence to VKA treatment within 4 weeks before the switch to rivaroxaban was compared with patient adherence to rivaroxaban by McNemar's test for paired samples.

The characteristics of participating physicians were compared to those of French cardiologists recorded by the French Direction for research, studies, evaluation and statistics using a Chi-squared goodness of fit test.

All statistical tests were two-sided and the conventional α-level of 0.05 was used for significance testing.

### Ethical considerations

Written informed consent was obtained from every included patient. The study was conducted according to the ethical principles of the Declaration of Helsinki and in accordance with Good Epidemiological Practices. Approvals from the French review boards “Comité consultatif sur le traitement de l’information en matière de recherche dans le domaine de la santé” and “Commission nationale de l’informatique et des libertés” were obtained.

## Results

From 5 April 2013 to 30 June 2014, 51 French cardiologists enrolled 422 patients switching anticoagulation from VKA to rivaroxaban of whom 405 composed the Per Protocol Set (PPS). A large majority of physicians were private practitioners (74.5%) and their geographical repartition was not different from that of the French cardiologists according to the French Direction for research, studies, evaluation and statistics.

### Patient characteristics

Demographic and clinical characteristics for the 405 included patients are shown in Table 1. The majority (84.4%) were aged over 65 years old (mean age was 74.8 (standard deviation = 9.0), 63.0% male) and in 90.3% AF was confirmed by a previous ECG. The mean time since first AF diagnosis was 6.1 (6.5) years. Atrial fibrillation was paroxysmal in 40.0%, persistent in 11.6%, permanent in 43.2% of patients and undisclosed in 5.2%. For 69.1% of patients AF was asymptomatic. The mean CHADS_2_ and CHA_2_DS_2_-VASc scores were 1.9 (1.1) and 3.4 (1.5) respectively and were ≥ 2 points in 58.5% and 87.2% of the cohort. The mean HAS-BLED score was 2.9 (1.2) and was ≥ 3 points in 56.8%.

**Table 1:** Demographic data and risk factors.

General characteristics, %	Total N = 405
Age, M (SD), year	74.8 (9.0)
Gender, male	63.0
BMI, M (SD), kg/m^2^	27.9 (5.0)
Obesity (BMI > 30kg/m^2^)	26.7
Hypertension	68.4
Diabetes mellitus	18.8
Heart failure	14.8
LVEF < 40%	8.1
Prior stroke / transient ischemic attack	15.3
Prior myocardial infarction	7.9
Prior systemic embolism	2.0
Prior VTE	6.9
Bleeding history	3.2
Patients taking antiarrhythmic drugs^a^	40.7
Patients with rhythm control strategy^b^	59.3
Risk scores, M (SD)	
CHADS_2_	1.9 (1.1)
CHA_2_DS_2_-VASc	3.4 (1.5)
HAS-BLED	2.9 (1.2)

^a^ Amiodarone and/or flecainide and/or sotalol and/or propafenone

^b^ Defined as intake of at least one antiarrhythmic drug, ablation, pacemaker or cardioversion within the last year

M (SD), mean (standard deviation); BMI, body mass index; LVEF, left ventricular ejection fraction; VTE, venous thromboembolism.

Before switching to rivaroxaban, most patients (87.4%) were treated with fluindione and received a median daily dose of 15 mg, the others were treated with acenocoumarol (6.2%) or warfarin (6.4%) and received a median daily dose of 3 mg and 4 mg respectively. The mean duration for previous VKA treatment before switching to rivaroxaban was 4.7 (5.4) years with a median duration of 3 years. The main reason for the switch was unstable INR (59.8%), patient’s decision (24.4%) and physician’s decision related to other reason than unstable INR (15.3%).

When starting their new treatment, 74.8% of patients were prescribed with 20 mg rivaroxaban, 24.4% with 15 mg and one patient with 10 mg. For two patients, the prescribed dose was not reported.

Overall, 96.5% of patients received concomitant medications, mainly angiotensin converting enzyme inhibitors/ angiotensin receptor blockers (60.5%), beta-blocker agents (50.1%), statins (41,0%), diuretics (28.4%), antiarrhythmic therapies (23.7% amiodarone, 11.9% flecainide, 4.9% sotalol and 0.7% propafenone) and digoxin (12.3%). About 7% of patients received antiplatelet agents (3.5% acetylsalicylic acid, 3.0% clopidogrel and 0.5% both acetylsalicylic acid and clopidogrel).

### Patient satisfaction

The ACTS (burdens and benefits) measures were completed by 99% of patients at baseline, 93% at 1 month, 89% at 3 months and 85% at 6 months.

**Fig 1:**
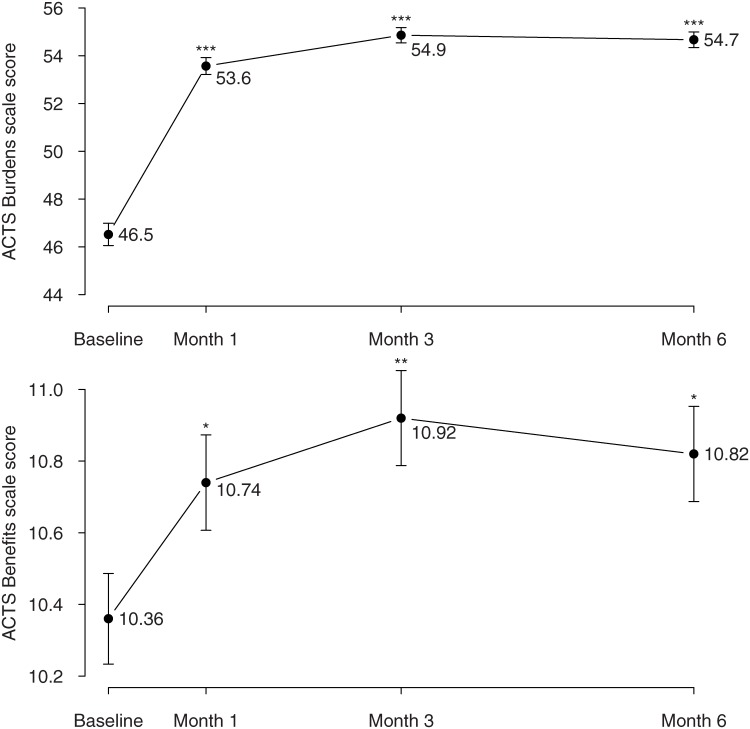
Means +/- standard errors of patient satisfaction with anticoagulant therapy measured by the Anti-Clot Treatment Scale (ACTS) over time. * p < 0.05, ** p<0.01, *** p < 0.001, Wilcoxon rank-sum test comparing baseline ACTS at baseline and ACTS after 1, 3 and 6 months for burden and benefit ACTS subscales separately.

The mean ACTS burdens and benefits scores significantly improved between baseline and month 3 (baseline burden scale: overall mean 46.5 vs. month 3: mean 54.9, p<0.001, effect size 0.89; baseline benefits scale: overall mean 10.4 vs. month 3: mean 10.9, p = 0.001, effect size 0.16; see Table 2). Compared to baseline, patients also reported satisfaction with the treatment at 1 and 6 months (burden scale: month 1 mean 53.6 p<0.001; month 6 mean 54.7 p<0.001; benefit scale: month 1 mean 10.7 p<0.05; month 6 mean 10.8 p = 0.02; see Fig 1). No difference in ACTS change was observed according to age, except for ACTS burdens score at baseline (patients ≤65 years old: mean 44.4 (10.3); patients aged between 65 and 75 years old: mean 47.6 (9.4), p = 0.03).

**Table 2:** ACTS Burdens scale and Benefit scale scores at baseline and 3 months.

ACTS, Mean (SD)	Baseline	3 months	Absolute change	p^a^	Effect size
	N = 401	N = 360	N = 360		
ACTS burdens	46.5 (9.3)	54.9 (6.1)	8.3 (8.9)	<0.001	0.89
ACTS benefits	10.36 (2.53)	10.92 (2.51)	0.41 (2.85)	0.001	0.16

^a^ Wilcoxon rank-sum test comparing baseline ACTS and ACTS after 3 months

### Patient quality of life

A slight improvement of quality of life at 3 months was observed for SF-36 and each sub-score by domain, but these changes did not reach the significant threshold of clinical relevance. Moreover, results observed at 1 and 6 months also showed no clinical improvement. Same results were observed regarding SF-36 scores according to age, although median SF-36 scores were lower (at each time point) in patients > 75 years old.

### Rivaroxaban continuation

**Fig 2:**
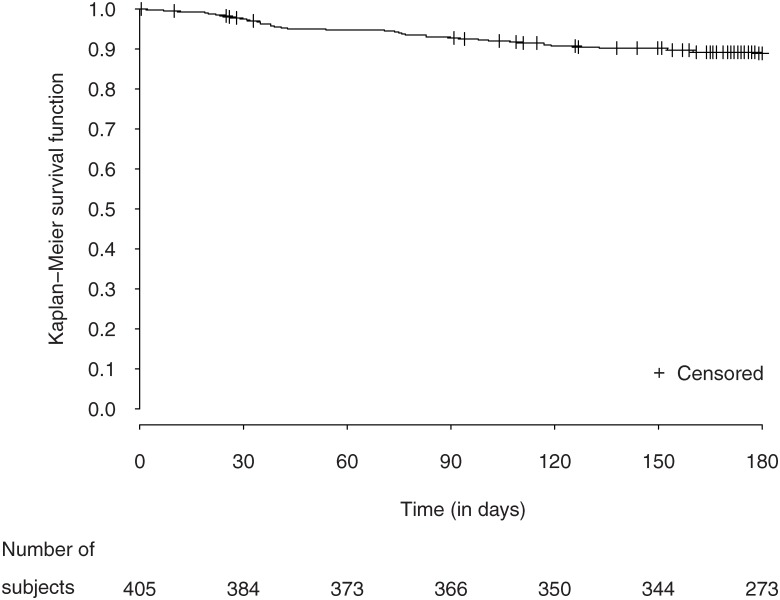
Continuation rate with rivaroxaban treatment over time.

The rate of treatment continuation per 100 patient-180 days was found to be 88.7% (95% CI 85.1–91.5) (see Kaplan Meyer curve, Fig 2). Rivaroxaban discontinuation was recorded for 53 patients. The percentage of patients who early discontinued rivaroxaban (within 90 days after treatment initiation) tend to be slightly higher in the subgroup of patients aged over 75 (4.4% and 8.3% at 30 and 90 days compared to 1.0% and 5.5% in the subgroup of patients aged under 75) but discontinuation rates at 6 months were similar irrespective of age (10.3 to 11.2%). The main reasons for treatment discontinuation were occurrence of an adverse event (69.8%), patient’ decision (17.0%) and other reasons (11.3%). Data were missing for 2 patients. For 14 participants the adverse events leading to rivaroxaban withdrawal was a bleeding event. Three patients switched to another anticoagulant treatment. The patients who discontinued rivaroxaban had a duration of VKA treatment before switching to rivaroxaban comparable to that of patients who did not discontinue rivaroxaban treatment (5.51 (6.04) years vs. 4.57 (5.27) years, p = 0.39).

### Patient adherence and investigator satisfaction

Patient adherence to rivaroxaban was compared with adherence to VKA assessed within 4 weeks before the switch to rivaroxaban. Adherence to rivaroxaban was significantly higher than to VKA treatment. According to patient’s interview, 4.7% of patients forgot to take their VKA treatment (sometimes, frequently or all the time) before switching to rivaroxaban, while 0.8% forgot to take rivaroxaban after 6 months of treatment (p<0.001). Results were similar for rivaroxaban adherence 1 and 3 months after initiation (respectively 98.2 and 97.5%, see Table 3).

**Table 3:** Adherence to treatment over time.

Treatment not taken during the last 4 weeks before baseline visit	VKA treatment	Rivaroxaban treatment		
	Baseline	Visit M1	Visit M3	Visit M6
N	405	390	365	355
Missing values	1 (0.2%)	1 (0.3%)	1 (0.3%)	1 (0.3%)
Never or rarely	385 (95.1%)	383 (98.2%)	356 (97.5%)	351 (98.9%)
Sometimes or frequently or all the time	19 (4.7%)	6 (1.5%)	8 (2.2%)	3 (0.8%)
p value^a^		0.02	0.04	0.001

^a^ McNemar's test for paired samples comparing VKA adherence and rivaroxaban adherence after 1, 3 and 6 months

Furthermore, after 6 months of treatment, physicians were satisfied or very satisfied with the treatment for 89.6% of patients. Satisfaction rates were similar throughout the study (84.1% and 90.7% after 1 and 3 months respectively).

### Safety and security profile

In subjects for whom an estimated glomerular filtration rate (eGFR) was calculated (with Cockcroft method) at baseline (n = 200; 49%), we observed that 4 subjects had a prescription of rivaroxaban 20 mg whereas their baseline eGFR was lower than 50 ml/min, and that 30 subjects had a prescription of rivaroxaban 15 mg while they had a eGFR >50 ml/min Thus, we identified 34 patients (17%) among those who had a eGFR calculated by the physician not prescribed with the appropriate dosage of rivaroxaban (either underdosing or overdosing).

During the study, a total of 195 adverse events (AE) were reported by 126 patients (30.7%): 81 patients (19.7%) experienced 106 AE related to treatment and 42 patients (10.2%) experienced 59 AE leading to treatment discontinuation. Forty-three serious adverse events (SAE) were reported, 11 being related to rivaroxaban. The most frequently reported AEs were gastrointestinal disorders (9%; mainly gingival and digestive bleeding), nervous system disorders (6.1%; mainly dizziness), respiratory, thoracic and mediastinal disorders (4.4%, mainly epistaxis), renal and urinary disorders (3.9%), general disorders and administration site conditions (3.2%), cardiac disorders (2.7%), musculoskeletal and connective tissue disorders (2.2%), skin and subcutaneous tissue disorders (2.2%).

Special attention was paid to bleeding events (BE), and more particularly to major BE (predefined as fall in hemoglobin ≥ 2 g/dl, or transfusion ≥ 2 units of packed red blood cells or whole blood, or occurrence in a critical organ [intra-cranial, intra-spinal, intra-ocular, pericardial, intra-articular, intra-muscular with a compartment syndrome, retroperitoneal], or death). Sixty-two BE were recorded (13 serious BE and 4 major), most of them being spontaneous bleeding (75.4%) and clinically overt (85.2%). Fifty-six BE were related to treatment (11 being serious BE) and 15 led to treatment discontinuation.

Three patients died (0.7%) during the study: a 62-year old female died after choking on food, a 73-year old male died from ventricular fibrillation and a 73-year old male died from a massive hemorrhagic stroke that was considered as possibly related to rivaroxaban.

## Discussion

The SAFARI study describes PRO evidence supporting that patient satisfaction with rivaroxaban therapy was high and significantly increased after the switch from VKA to rivaroxaban.

Patient-reported outcome tools such as the ACTS questionnaire are becoming increasingly important in patient care, policy-making, and prescription. Treatment satisfaction should be measured using instruments that have been both specifically designed for this purpose and psychometrically validated [13]. The ACTS fulfills these requirements and aims to delineate the burdens and benefits associated with anticoagulation treatment [9]. Both the ACTS Burdens and ACTS Benefits scales consistently satisfied traditional reliability and validity criteria across multiple language populations (including French language), supporting the fact that it is a clinically useful patient-reported instrument of satisfaction with anticoagulant treatment in clinical trials [10]. The SAFARI study has provided robust data on the patient-reported burden and benefit of the switch from VKA to rivaroxaban in this patient population.

### Clinical implications

Our results are in line with EINSTEIN study, an open-label randomized clinical trial performed in deep vein thrombosis [9] and pulmonary embolism [14] population that demonstrated a similar improvement of ACT score in rivaroxaban group compare with warfarin group. In EINSTEIN study the population was selected and accepted to participate in a therapeutic trial thus being more likely to adhere to treatment and to be satisfied with the new drug than patients in real-world practice. Our study found a higher patient satisfaction with rivaroxaban than with VKA in a non-selected population in real-life condition. SAFARI is one of the first studies to demonstrate an improvement in patient satisfaction with rivaroxaban in AF population.

Moreover, the improvement of patient satisfaction with rivaroxaban was observed as soon as 1 month after the switch from VKA and remained throughout the follow-up. This is consistent with EINSTEIN-PE in which the patient-perceived benefits of rivaroxaban became apparent from day 15 onwards. These results show that improvement appears soon after the treatment switch and is sustained throughout the treatment period.

As in EINSTEIN study, increases in effect size in ACTS Burdens scores with rivaroxaban were higher over time compared with ACTS Benefits scores. Improvement of satisfaction with rivaroxaban may be related to an easier use including few drug interactions, a lack of dietary restrictions and no requirement for regular coagulation monitoring that makes VKA difficult to manage in routine clinical practice. Improvement of patient satisfaction may also be related to the good safety profile. Our results are consistent with the Star study that shows AF patient satisfaction increases with rivaroxaban compared with VKA with a shorter follow-up and with a 6-response Likert rating scale [15].

Patient satisfaction with oral anticoagulation treatment is essential because of the strong relationship between patient satisfaction and adherence to treatment [16,17].

In our study, adherence to rivaroxaban was higher than to VKA treatment when considering adherence to VKA treatment within 4 weeks before switch to rivaroxaban (98% vs. 95%, p<0.05). High medication adherence to novel oral anticoagulant (NOAC) has already been shown; 83% with rivaroxaban in thromboprophylaxis of knee and hip surgery (percentage of patients that completed all prescribed doses) [18] 2014 92% with rivaroxaban in AF population (no missed dose in the previous week) [19] and 72% with dabigatran in a AF cohort of Veterans Affairs hospitals (proportion of days covered > 80%) [20]. Reduced treatment burden and regimen complexity may favor better adherence to rivaroxaban and may therefore contribute to increasing patient adherence and improving stroke prevention in AF patients [9]. Furthermore drug persistence for patients treated with rivaroxaban was high—treatment continuation > 88.7% after 6 month—and appears higher than for VKA treatment as recently demonstrated in a “real world” cohort study (i.e., 63.6% for VKA vs. 79.2% for NOAC after one year) [4]. Drug persistence to oral anticoagulation is an important topic because of stroke prevention [21] and there is a need for daily care drug persistence data which may differ from phase III trial results. In our study, discontinuation rate for rivaroxaban (13.1%) was similar to that observed in a Dresden non-interventional oral anticoagulation registry (13.6%) [21] and close to that of Xantus study in which the discontinuation was 20% after 1 year [22].

In contrast, no improvement on quality of life was observed after 6 months of follow-up. Health-related quality of life (HRQoL) in AF patients is mainly related to their level of disability. So, regarding the domains explored by the SF-36 scale (mental health, emotion, social functioning, vitality, physical functioning…), the benefit of rivaroxaban on quality of life, if it exists, may be mainly due to more prevented intracranial events resulting in less disability, than by an easier use. In our study few bleeding events and no ischemic event were observed during the 6-month follow-up. Our results are in line with the RELY trial that compared another NOAC with warfarin in a randomized controlled trial in AF population. Neither dabigatran nor warfarin had a relevant impact on quality of life over the course of 1 year, as assessed by the EQ-5D score [23]. Furthermore, it should be noted that the self-assessed patient health and quality of life, as assessed using the SF-36 questionnaire, were already good at baseline and throughout the study, thus little improvement could be observed for quality of life after 6 months of treatment. These results indicate that a specific questionnaire like ACTS is more relevant than SF-36 or EQ-5D to assess medication satisfaction of a new drug like NOAC. Moreover, this information is also important for cost-effectiveness models in the context of health technology assessments of new drugs.

Rivaroxaban was generally well tolerated, with a good overall safety profile. Overall, approximately 30% of patients experienced an AE, with 20% reporting AEs related to rivaroxaban, and 8% experiencing an SAE. Less than 3% of the patients experienced a serious bleeding event related to rivaroxaban.

Regarding condition of use of rivaroxaban, we observed that about 17% of the patients were not prescribed with the appropriate dosage based on their eGFR, most of them (more than half) receiving insufficient dosage (15 mg instead of 20 mg). Thus, in the whole study population, we identified about one third of patients prescribed with rivaroxaban 15 mg instead of 20 mg. This proportion may even be higher considering the fact that eGFR was only reported for less than half of the patients at baseline even though creatinine measurement is highly recommended by French Health Authorities before rivaroxaban inception. These observations highlight drug misuse thus pointing the need for promoting proper use and ensuring safe use of the product.

### Strengths and limitations

The major strength of the study is the widely data collection on a large sample of consecutive patients with AF in real life condition. Our sample was mainly composed of 65–85 year patients which reflects the real-world AF population as shown in epidemiological study on AF [24–28]. This observational study provides insight about the characteristics of patients and their satisfaction with treatment under real-life clinical practice conditions that cannot be provided by controlled clinical studies. Patient satisfaction was measured by the ACTS questionnaire that has been shown to meet widely accepted reliability and validity criteria [9,10].

This study has also several important limitations. Patients included in the study were exclusively patients that had undergone a switch from VKA to rivaroxaban treatment, thus introducing a potential bias in patient selection. However, the switch decision is often taken by the cardiologists mainly because of higher net benefit of NOAC over VKA, unstable INR, bleeding or thromboembolic complications and repeated falls rather than because of dissatisfaction of patients [21]. Although patient satisfaction results show that patients are more at ease with the use of rivaroxaban, the 6 months observation period is actually short and could have been longer. Treatment adherence could have been overestimated because it was based on investigator judgment following interview of the patient and patient reports. The overall assessment of adherence could have been improved by the implementation of better quantification processes, and the use of ‘adherence scale’ [29]. Finally, no control group was used in this study, therefore we cannot compare the complications that occurred with Rivaroxaban with the complications that occurred with vitamin K antagonists. Meanwhile in a previous, large-scale, event-driven, non-inferiority study, patient satisfaction of rivaroxaban treatment given for the prevention of deep vein thrombosis and pulmonary embolism was compared directly with VKA treatment [9].

## Conclusion

In patients with AF in real-life condition, the switch from VKA to rivaroxaban for the prevention of stroke and systemic embolism improved patient satisfaction from 1 month up to 6 months after rivaroxaban initiation. A high persistence and high adherence to rivaroxaban was also observed with a good safety profile. The increase of patient satisfaction from baseline to month 3, with little further change to month 6, indicates a significant improvement to an extent not previously demonstrated in clinical trials in AF population.
